# Biomarkers of seaweed intake

**DOI:** 10.1186/s12263-019-0648-4

**Published:** 2019-08-14

**Authors:** Muyao Xi, Lars O. Dragsted

**Affiliations:** Department of Nutrition, Exercise and Sports, University of Copenhagen, Copenhagen, Denmark

**Keywords:** Biomarkers of food intake, Food exposure marker, Dietary assessment, Seaweed, Macroalgae

## Abstract

Seaweeds are marine macroalgae, some of which are edible. They are rich in specific dietary fibers and also contain other characteristic biological constituents. Biological activities have been investigated mainly in animal studies, while very few results are available from human studies. Biomarkers of food intake (BFIs) specific to seaweed could play an important role as objective measurements in observational studies and dietary intervention studies. Thus, the health effects of seaweeds can be explored and understood by discovering and applying BFIs.

This review summarizes studies to identify candidate BFIs of seaweed intake. These BFIs are evaluated by a structured validation scheme.

Hydroxytrifuhalol A, 7-hydroxyeckol, C-O-C dimer of phloroglucinol, diphloroethol, fucophloroethol, dioxinodehydroeckol, and/or their glucuronides or sulfate esters which all belong to the phlorotannins are considered candidate biomarkers for brown seaweed. Fucoxanthinol, the main metabolite of fucoxanthin, is also regarded as a candidate biomarker for brown seaweed. Further validation will be needed due to the very limited number of human studies.

Further studies are also needed to identify additional candidate biomarkers, relevant specifically for the red and green seaweeds, for which no candidate biomarkers emerged from the literature search. Reliable BFIs should also ideally be found for the whole seaweed food group.

**Electronic supplementary material:**

The online version of this article (10.1186/s12263-019-0648-4) contains supplementary material, which is available to authorized users.

## Introduction

Seaweeds or macroalgae, including species of brown, red, and green seaweeds, have been consumed as food in East Asia for a long time, but with more limited use in Europe and North America, except for the use of certain constituents as additives [1]. Recent interest in manufacturing and consuming high-value food products derived from seaweeds in Western countries is fueled by their proposed health benefits as well as by the introduction of Asian foods. Seaweeds may supply several nutrients and have been proposed to promote health and counteract a wide range of conditions and diseases, such as obesity, oxidative stress, hypertension, digestive problems, thrombosis, and even cancer [2–5]. However, this builds largely on very limited evidence from animal and in vitro studies. There are also a few trials with seaweeds or seaweed preparations in humans investigating effects on blood pressure [5, 6], appetite [7, 8], inflammation [9], and insulin response [10]. These health effects may originate from a variety of seaweed compounds, such as soluble fiber and carotenoids [6, 11–15]. Only one study has so far shown which compounds can be used as biomarkers of seaweed intake in overweight or obese subjects. In addition, no study has so far firmly documented longer-term effects of seaweed intake in human trials.

In order to explore the relationship of seaweed intake with human health, it is important to measure intake accurately and it is therefore of importance to find specific compounds related to seaweed intake in general or to intake of any certain type of seaweed. Intake assessments in human studies to date largely depend on dietary assessment instruments such as food frequency questionnaires (FFQ) or 24-h recalls (R24h). These instruments are prone to recall bias and systematic errors [16]. Lack of compliance is also common in dietary intervention studies. Thus, it is difficult to evaluate the relationship between health effects and seaweed intake. Well-validated biomarkers of food intake (BFIs) may provide more objective estimates of actual intake.

The objectives of this review are (1) to summarize information from the scientific literature related to compounds that may be currently used as, or considered as, candidate biomarkers for seaweed consumption [16] and (2) to provide systematic validation of the candidate BFIs based on recent methodology [17].

## Materials and methods

### Defining the food group

Seaweeds are not well-defined in biological terms but cover largely the macroalgae. The macroalgae contain species belonging to several different phyla consisting of red, green, or brown algae and in some cases are considered to include also the prokaryotic photosynthetic cyanobacteria. Within the food group of seaweeds, there is a corresponding subdivision into three food subgroups, the red, brown, and green seaweeds, each with a large number of species. Although there are differences within and between these 3 subgroups of seaweed, it is considered of interest to find candidate biomarkers of the overall seaweed group as well as for each subgroup. Within each subgroup, there are many species that are likely to vary considerably in composition; however, the present review will not emphasize the detail of species or of varieties within species because little is known at this level of detail.

### Primary literature search for relevant BFIs for seaweed intake

Original research papers were searched within three databases (Scopus, PubMed, and the ISI Web of Knowledge). The search made use of combinations of several search terms with inclusion and exclusion criteria as keywords, as described for the BFIRev literature search procedure [16]: (biomarker* OR marker* OR metabolite* OR biokinetics OR biotransformation OR pharmacokinetics OR bioavailability OR ADME) AND (intake OR meal OR diet OR ingestion OR consumption OR eating OR drink* OR administration) AND (human* OR men OR women OR patient* OR volunteer* OR participant*) AND (urine OR plasma OR blood OR serum OR excretion OR hair OR toenail OR feces OR fecal water) AND (seaweed OR macroalgae). The field of each database used as default is [Article Title/ Abstract/ Keywords] for Scopus, [All Fields] for PubMed, and [Topic] for ISI Web of Science. The search for papers on seaweed intake biomarkers was covering all papers published up until March 2019 but was restricted to papers in the English or Chinese language. Additional papers were acquired from reference lists of included papers after filtering and from reviews, books, or online databases (Google Scholar). Exclusion criteria avoided papers dealing only with effects of ingested seaweed on diabetes and other diseases, plasma lipids, organ damage, anti-oxidation, and other articles not relevant to intake biomarkers; animal studies were also largely excluded unless they contain information on unique seaweed-derived compounds. Papers on contaminants and mineral contents of seaweed were also excluded from the search.

### Secondary search—evaluation of putative BFIs

After identification of potential candidate BFIs from the primary literature search, a second search step was performed to evaluate the specificity of each putative BFI for seaweed intake. The second search was carried out by replacing (seaweed OR macroalgae) with (“the name and synonyms of the potential candidate biomarker” OR “the name and synonyms of potential candidate biomarker class”). In addition to the online databases listed above, hmdb [18] and foodb [19] were searched for each putative BFI in order to ascertain that they have not been measured previously in other foods. The full list of putative BFIs is shown in Table 1. Plausibility of the BFIs, i.e., the evaluation of whether the putative marker compound is likely to be uniquely present in seaweeds, was decided based on the secondary search; the final list of plausible BFIs are shown as the top 7 items of Table 2.

**Table 1:** List of studies reporting putative biomarkers for brown seaweed consumption

Dietary factor	Study design	Number and age of subjects	Analytical method	Sample type	Discriminating metabolites	Primary reference(s)
Brown seaweed (food-grade seaweed capsule) 400 mg containing 101.89 mg polyphenol	Single oral dose intervention study with no control group	12 women and 12 men (aged 18–65 years)	Plasma ((RP)-HPLC-DAD), urine (RP-HPLC-DAD and HPLC-MS)	Hydrolyzed urine	Hydroxytrifuhalol A, 7-hydroxyeckol, C-O-C dimer of phloroglucinol	[20]
				Hydrolyzed plasma	Data not shown in the paper	
Brown algae (*Ascophyllum nodosum*) capsule 400 mg containing 107.3 mg phlorotannin	A 24-week randomized, double-blind, placebo-controlled crossover trial	39 men and 41 women (aged 30–65 years)	UHPLC-HR-MS HPLC-DAD	Urine	Pyrogallol/phloroglucinol sulfate, hydroxytrifurahol A-glucuronide, dioxinodehydroeckol glucuronide, C-O-C dimer of phloroglucinol sulfate, diphlorethol sulfate, fucophloroethol glucuronide	[21]
				Plasma	No markers found	
10 ml kombu extract containing 31 mg fucoxanthin	Single oral dose intervention study with no control group	10 males and 8 females (aged 22–63 years)	HPLC-UV-VIS	Plasma	Fucoxanthinol	[22]
Stir-fried wakame containing 6.1 mg fucoxanthin	1 week dietary intervention study with no control group	3 women and 2men (aged 30–50 years)	(SPE)-HPLC-DAD	Plasma	Fucoxanthinol	[23]
Astaxanthin from a supercritical CO_2_ extract of *Haematococcus pluvialis* (green seaweed) as a softgel capsule (0, 2, 8 mg)	A 8-week double-blind, placebo-controlled study	42 females (aged 20–23 years)	RP-HPLC-DAD	Plasma	Astaxanthin	[24]
*Haematococcus pluvialis* extract. 12 soft capsules, each containing 4 mg of astaxanthin, total 48 mg	Single oral dose intervention study with no control group. Two females and five non-smoking males received the capsules 2 h in advance of a meal, the others took them right after the meal.	15 males (7 smokers) and 5 females (aged 18–60 years)	HPLC-UV-VIS	Serum	Astaxanthin	[25]
Green alga + lipid to be formulation contained 40 mg astaxanthin	Open parallel design dividing into 4 groups on average for 4 days	32 males (aged 20–46 years)	HPLC-VIS	Plasma	Astaxanthin	[26]
100 mg astaxanthin with olive oil and cereals for 3 days	Single dose intervention study with no control group	3 males (aged 37–43 years)	HPLC-UV-VIS	Plasma	Astaxanthin	[27]
Average 50 sheets of nori (average 200 g β-carotene) for 5 months	Sequential intervention	One 22-year-old female	/	Serum	β-carotene	[28]

Abbreviations: *DAD* diode array detector, *HPLC* high-performance liquid chromatography, *MS* mass spectrometry, *RP* reverse phase, *SPE* solid-phase extraction, *UHPLC-HR-MS* ultra-high-performance liquid chromatography–high-resolution mass spectrometry, *UV-VIS* ultraviolet-visible spectroscopy

**Table 2:** Summary of the putative biomarkers of seaweed intake, including reasons for inclusion or exclusion from the final list of candidate biomarkers

Food item	Metabolites	Biofluid locations	Reason for inclusion and exclusion	Selected for further systematic validation as BFIs
Brown seaweed	Hydroxytrifuhalol A	Hydrolyzed urine	Specificity and suitable post-prandial kinetics	Yes
Brown seaweed	7-Hydroxyeckol	Hydrolyzed urine	Specificity and suitable post-prandial kinetics	Yes
Brown seaweed	C-O-C dimer of phloroglucinol	Hydrolyzed urine	Specificity and suitable post-prandial kinetics	Yes
Brown seaweed	Dioxinodehydroeckol glucuronide	Urine	Specificity and suitable post-prandial kinetics	Yes
Brown seaweed	Diphlorethol sulfate	Urine	Specificity and suitable post-prandial kinetics	Yes
Brown seaweed	Fucophloroethol glucuronide	Urine	Specificity and suitable postprandial kinetics	Yes
Brown seaweed	Fucoxanthinol	Plasma	Quite specific, existing in many other marine foods but much lower than that in brown seaweeds	Yes
Brown seaweed	Pyrogallol sulfate	Urine	Unspecific, also a metabolite found after intake of tea, mango, berry fruits, nuts and red wine.	No
Brown seaweed	Phloroglucinol sulfate	Urine	Unspecific, also a metabolite found after intake of grape. Phloroglucinol is also a drug.	No
Brown seaweed	Fucoxanthin	None	Only reported in seaweed and at low levels in some crustaceans, not yet detected in human plasma	No
Green seaweed	Astaxanthin	Serum	Unspecific, existing in many other marine foods	No
Green seaweed	Astaxanthin	Plasma	Unspecific, existing in many other marine foods	No
Red seaweed	β-carotene	Serum	Unspecific, existing in many fruits and vegetables	No

### Validation criteria of candidate BFIs

The candidate BFIs were validated according to a set of criteria published recently [17]. There are 8 topics in this validation system (Additional file 1: Text S1) relating to aspects of analytical performance, applicability in nutrition research, and various aspects of biological validity, which were answered for each candidate BFI in Table 3.

**Table 3:** Overview of the current level of validation of the candidate biomarkers of seaweed intake

Food item	Metabolites	Biofluid locations	Questions^a^							
			1	2	3	4	5	6	7	8
Brown seaweed	Hydroxytrifuhalol A	Hydrolyzed urine	Y	U	Y	U	U	U	U	U
Brown seaweed	7-Hydroxyeckol	Hydrolyzed urine	Y	U	Y	U	U	U	U	U
Brown seaweed	C-O-C dimer of phloroglucinol	Hydrolyzed urine	Y	U	Y	U	U	U	U	U
Brown seaweed	Dioxinodehydroeckol glucuronide	Urine	Y	U	Y	U	U	U	U	U
Brown seaweed	Diphlorethol sulfate	Urine	Y	U	Y	U	U	U	U	U
Brown seaweed	Fucophloroethol glucuronide	Urine	Y	U	Y	U	U	U	U	U
Brown seaweed	Fucoxanthinol	Plasma	Y	U	Y	U	U	U	U	U

^a^The criteria are 1, plausibility; 2, dose response; 3, time response; 4, robustness; 5, reliability; 6, stability; 7, analytical performance; 8, reproducibility. The full text of questions Q1–Q8 is reported in Additional file 1: Text S1. Possible answers are Y (yes, the criterion is fulfilled for at least some use of the biomarker), N (no, the criterion has been investigated but it was not fulfilled), or U (uncertain, the criterion has not been verified or data is not available). The questions are based on the criteria outlined by Dragsted et al. [17]

## Results

**Fig. 1:**
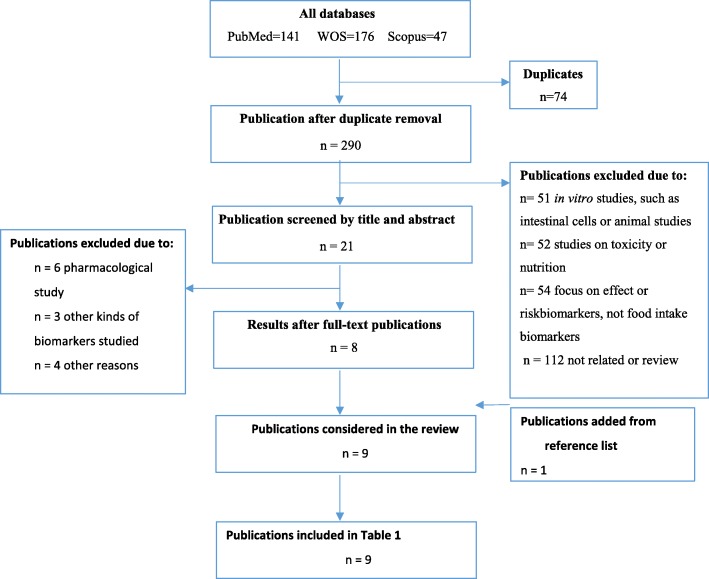
Flow diagram of systematic literature search according to the BFIRev methodology. The three databases PubMed, SCOPUS, and Web of Science were searched as outlined in the “Methods” section. After duplicate removal, titles were screened to select papers for further screening of abstracts. Abstracts were screened for selecting full-text papers, and reference lists of the selected papers were screened for additional relevant papers. During the process, most titles and abstracts were excluded for reasons outlined in the side boxes. A total of nine relevant references were included in the review

The original search process retrieved 364 research papers, of which 290 remained after excluding duplicates. Subsequently, the number decreased to 21 eligible papers after screening titles and abstracts. The other 269 papers were eliminated because they were not relevant in relation to seaweed BFIs, mainly because they were not concerned with seaweed constituents or mentioned only contents of common nutrients in seaweeds. The twenty-one remaining eligible full-text articles were evaluated for relevant content about BFIs. The reference lists were additionally checked to acquire more relevant papers. As a result of this selection process, 8 papers were identified from the database search and 1 paper from reference lists for further review (Fig. 1 and Additional file 1: Table S1). The relevant information from the selected 9 papers is extracted into Table 1. The studies included four single oral dose intervention studies, one unblinded short-term and one unblinded long-term intervention study; one short-term, double-blind, placebo-controlled and one short-term open-label parallel study; and one long-term randomized, double-blind, placebo-controlled crossover trial. However, no observational studies were found to use BFIs.

In a secondary search, we carried out a web-database check and a literature search for compounds from Table 1 to evaluate whether these compounds are specific to seaweed or might exist in other foods. Non-specific biomarkers are listed as the last 6 items in Table 2, including relevant exclusion criteria. Additional file 1: Table S1 lists the source of primary literature and information collected from these studies.

Hydroxytrifuhalol A, 7-hydroxyeckol, C-O-C dimer of phloroglucinol, diphloroethol, fucophloroethol, dioxinodehydroeckol, and/or their glucuronides or sulfate esters (Additional file 1: Figure S1) were selected as candidate BFIs for brown seaweed because of their apparent specificity and their suitable postprandial kinetics [20, 21]. Fucoxanthinol, a metabolite of fucoxanthin, has also been found in plasma from a study after only a single oral dose of brown seaweed, making it interesting also as a candidate short-term biomarker [22].

Some markers were not added to Table 2 although they are known to be present in seaweed. These include fucoxanthin [22, 23], siphonaxanthin [29], astaxanthin [24, 25, 27, 30], and beta-carotene [28]. The first two of these have not so far been observed in human blood and therefore cannot be validated. The last two are abundant in many other foods and are therefore not even plausible as specific BFIs for seaweed. Another 2 markers (pyrogallol sulfate [31], phloroglucinol sulfate [32]) were excluded from Table 2 since they are also metabolites of many other foods, and phloroglucinol is also potentially used as a drug [33].

Further experimental work needs to be done to find BFIs for intake of green and red seaweed while the 7 compounds in Table 3 may also be considered as candidate BFIs for seaweed in general, either alone or in combination. While these compounds are candidate BFIs for intake of brown seaweed, their specificity to the brown seaweeds is not well documented and they may potentially also be useful as BFIs to assess the intake of the whole seaweed subgroup of marine foods. This would need further studies of the presence of these compounds in red or green seaweeds.

The seven candidate BFIs were assessed by the validation criteria published recently [17] as discussed below and shown in the legend of Table 3 and in Additional file 1: Text S1. The seven compounds are promising BFI candidates meeting several of the published validation criteria; however, we still lack information on their robustness, e.g., evidence from cross-sectional studies that these compounds are only observed after intake of seaweed and not after any other foods. In some cases, combinations of unspecific markers, which each overlap with a few other foods may provide very good specificity [34] as qualitative markers (e.g., seaweed intake yes/no) and such unspecific markers are therefore reasonable to retain even if the single compounds fail validation. The quantitative dose-response relationship of BFIs in human samples will also be needed to provide evidence for their use in quantification of seaweed intake. Currently, none of the candidate BFIs is fully validated for estimation of seaweed intake by all the validation criteria. Therefore, more experimental and observational studies need to be done on this food group to validate the current candidate biomarkers or their combinations.

## Discussion

Seaweeds have an ancient history as foods in certain regions and cuisines and extraction of hydrocolloids from seaweed as food additives dates back several centuries [35]. Seaweed consumption has been investigated scientifically only over the past 20–30 years, primarily focusing on chemical constituents of potential benefit to food science or for their health effects or risk to consumers. Many seaweed studies in the area of food chemistry focus on compounds, which have potential health benefits, such as polyphenols [20] or they are concerned with the nutritional compounds in seaweed, such as iodine [36], or with contaminants, such as arsenic [37]. The current review has evaluated potential seaweed BFIs; components used as food additives, nutrients, and contaminants were excluded as BFIs because they are not expected to be specific to seaweeds.

### Brown seaweed biomarkers

#### Phlorotannins

**Fig. 2:**
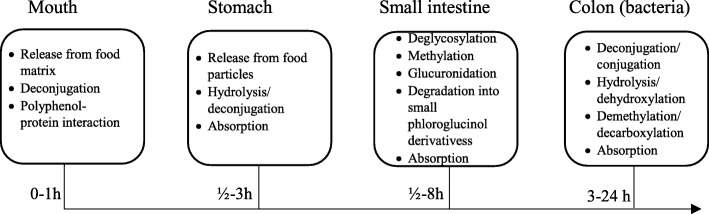
Summary of seaweed polyphenol ADME. The main factors affecting absorption, metabolism, distribution, and excretion of seaweed polyphenols in different segments of the gastrointestinal tract are listed in each box. Redrawn based on [53]

Brown seaweeds have a high content of polyphenolic compounds. The concentrations of phenols in brown seaweed species are higher than in red or green seaweeds [38, 39] or in other marine plants [40]. The main group of polyphenols in the marine brown seaweeds is phlorotannins, a type of phenolic constituents common to seaweeds and mainly found in brown species such as *Cystoseira nodicaulis*, *Cystoseira tamariscigolia*, and *Fucus spiralia* [41–46]. Phlorotannins are a highly diverse group, comprising a series of phloroglucinol derivatives [45]. There are currently very few human studies on the metabolism and bioavailability of seaweed phlorotannins. Therefore, there is a lack of human studies to compare the bioavailability of polyphenols between different seaweed species. Corona et al. [20] found that some oligomeric phlorotannins can be detected in seaweed polyphenol extract as well as in extracts which have been digested and fermented in vitro. The raw polyphenol extracts were provided as capsules to 24 volunteers while collecting blood and urine samples up till 24 h, respectively [20]. There are generally two concentration peaks both in plasma and urine, in which the first one is at 1–2 h in plasma as well as in urine and the second one is at 4–8 h in plasma and 8–24 h in urine following a polyphenol-rich meal [47]. In this study, the majority of phlorotannin metabolites appeared at 6–24 h, indicating initial small intestinal absorption of less-complex polyphenols and subsequent large intestinal absorption after fermentation of more-complex polyphenols into smaller molecules; enterohepatic circulation may also wholly or partially explain the observation. These polyphenols could possibly be seen as potential BFIs for recent seaweed intake. A range of phlorotannin metabolites were identified in plasma and urine separated by HPLC, especially hydroxytrifuhalol A, 7-hydroxyeckol, and the C-O-C dimer of phloroglucinol, identified only in urine; the structures were further confirmed by LC-MS/MS in the negative ionization mode, thereby confirming also some of the pathways of gut microbial phlorotannin degradation [20]. In addition, two unknown pseudomolecular ions (m/z 289 and 377) corresponded to metabolites present both in urine after intake of extracts and in vitro after simulated GI digestion and fermentation of extracts. Therefore, these two ions can be regarded as clues for further identifications [20]. Another study [21] also reported that the phloroglucinol derivatives, pyrogallol and phloroglucinol sulfates, dioxinodehydroeckol glucuronide, diphlorethol sulfate, and fucophloroethol glucuronide, can be found in human urine after seaweed intake. Pyrogallol and phloroglucinol sulfates would not be considered as plausible biomarkers of seaweed, although they can be measured in urine after seaweed intake. Pyrogallol sulfate is a common microbial phenolic metabolite, which is also found in both urine and plasma after intake of tea, mango, berry fruits, nuts, and red wine [48–52]. Therefore, it is not a specific metabolite after seaweed consumption. Phloroglucinol sulfate is also a metabolite after intake of several other foods, such as grape [32]. It is reported as a drug as well [33]. Thus, phloroglucinol sulfate is not specific enough to be regarded as a biomarker of seaweed intake. Figure 2 shows that seaweed phlorotannin polyphenols may undergo gastric digestion, small intestinal deglucosylation, and absorption to be metabolized by phase II enzymes in line with other plant polyphenols. Some phlorotannin metabolites can be detected in the plasma and urine between 0 and 8 h after seaweed intake. Unabsorbed phlorotannins will reach the large intestine where enzymes of the gut microbiota may play an important role to metabolize phlorotannins into smaller phloroglucinol-related compounds, leading to a second phase of absorption of the majority of metabolites with peaks at 6–24 h in plasma and excretion between 8 and 24 h in urine. In plasma and urine samples, which have not been treated with glucuronidase or sulfatase, a number of the phase II metabolites can appear [20, 47, 54]. In the study by Corona et al., there were substantial differences among volunteers in their pattern and extent of phlorotannin metabolism [20]. The total level of phlorotannins and their metabolites ranged from 0.01 to 7.8 μg/ml and from 0.15 to 33.5 μg/ml quantified as phloroglucinol equivalents in urine and plasma, respectively [20], while the range extended from 0.13 to 522.09 μg/ml quantified as phloroglucinol equivalents in urine in another study [21]. The large inter-individual differences have been attributed to variation in the microbiota composition in the colon and to differences in expression of phase II metabolizing enzymes [47, 55], suggesting large inter-individual variation in metabolite kinetics and, consequently, in their potential usefulness as BFIs for quantitative intake assessment. It is uncertain whether additional metabolites are formed but not yet identified or what specific microbial functionality is needed to degrade these particular compounds.

One of the papers has measured some of the metabolites in plasma (with the data not shown) [20] while the other paper did not observe any polyphenol metabolites in the plasma metabolome [21]. Both studies provided the postprandial time-course kinetics of measured metabolites in urine or plasma without including dose-response relationships. Some additional factors [56] may also affect ADME and need to be taken into consideration, including the effect of the food matrix, of cooking, or/and of processing on phlorotannin contents. Another consideration is the lack of commercially available standards for the phlorotannin-derived candidate BFIs. Phlorotannins may be quantified as phloroglucinol equivalents using phloroglucinol as standard in a colorimetric assay [57], but the method is imprecise and may not provide the same color reaction for conjugated metabolites. Because hydroxytrifuhalol A, 7-hydroxyeckol, dioxinodehydroeckol glucuronide, diphlorethol sulfate, fucophloroethol glucuronide, and C-O-C dimer of phloroglucinol are phlorotannin oligomers, most of them are metabolized in the colon by the microbiota, possibly into smaller phenolics. More work still needs to be done to identify these metabolites and to validate their specificity as seaweed BFIs.

#### Fucoxanthin and fucoxanthinol

Brown seaweeds are the exclusive source of fucoxanthin production in the food chain. Fucoxanthin, the main carotenoid in brown seaweeds, acts as a component of the light-harvesting complex for photosynthesis and photoprotection, which colors brown algae and the compound does not exist in red or green algae [58–61].

The fucoxanthin content of various brown seaweeds has been summarized based on several studies showing a reported interval of 0.3–3.7 mg fucoxanthin per gram dry weight of algae [23, 62]. Fucoxanthin and its metabolites are also present in many species of marine mollusks, such as clams (e.g., *Corbicula fluminea*), mussels (e.g., *Mytilus edulis*), chitons (e.g., *Chiton magnificus*), sea snails (*Haliotis* spp.), and possibly other bivalves because their main food source consists of seaweeds and diatoms [63–66]; the metabolites have been identified based on data from spectroscopic and chromatographic analyses. The specificity of fucoxanthin or its metabolite, fucoxanthinol, as intake biomarkers specific for consumption of seaweeds therefore needs some additional consideration; some studies show that the concentration of fucoxanthin ranges from 0.0045 to 0.0795 mg per 100 g edible part of marine mollusks, which equals 0.27–6.59 μg per gram dry weight [65, 67]. Therefore, the content of fucoxanthin in seaweed is much higher than that in marine mollusks. So if brown seaweeds are part of the diet, they are the more important dietary source of fucoxanthin compared to other marine foods. However, fucoxanthin cannot be detected in plasma, even after brown seaweed consumption [22, 23], most likely because it is extensively hydrolyzed into fucoxanthinol.

Fucoxanthinol, the hydrolyzed, deacetylated metabolite of fucoxanthin, has been detected in human plasma by HPLC. The compound has also been found in the intestinal tract or intestinal cells. Two studies [22, 23] have shown that the concentration of fucoxanthinol in human plasma is around 0.8 (SD 0.4) nM after the final day of a 1-week seaweed intervention providing 6.1 mg fucoxanthin from 6 g dried wakame per day but as much as 44.2 nM 4 h after a meal containing 31 mg fucoxanthin from 10 ml of kombu extract. According to these 2 human studies, we may conclude that the concentration of fucoxanthinol in plasma was associated with the duration of the period of intake and the concentration of fucoxanthin provided in the food. A review [63] illustrated that a similar metabolic pathway for fucoxanthin exists in marine animals, leading to the formation of fucoxanthinol, although there may be minor differences between species. Fucoxanthinol in particular was present in tissues of some marine mollusks as an intermediate metabolite of fucoxanthin. In general, animals such as clams cannot synthesize carotenoids by themselves de novo, leaving two possibilities. One is the direct accumulation from their feed, and the other is that partial modification of ingested carotenoids takes place through metabolic reactions within the clams. Some studies also report that fucoxanthinol can be extracted from the edible part of marine mollusks ranging from 0.09 to 15.52 μg per gram dry weight [64, 65, 67]. Thus, the concentration in marine mollusks is much higher than that in human plasma. However, due to the scarcity of data, we still need further studies to evaluate whether this metabolite could be a candidate biomarker for brown seaweed. Maoka et al. [64] also concluded that the major food source of bivalves (clams) are diatoms (unicellular algae), which would include several species that produce fucoxanthin. Fucoxanthin can be further metabolized into halocynthiaxanthin 3’-acetate, mytioxanthin, crassostreaxanthin A, and amarouciaxanthin A in bivalves. Amarouciaxanthin A has also been detected as a hepatic metabolite in mice [68]. However, there are no human studies reporting any of these metabolites. It is therefore important to consider the relative importance of seaweed and other seafoods (e.g., shellfish) to the intake of marine xanthophylls and their metabolites. More human studies will be needed in order to find more reliable biomarkers to discriminate between the marine plants and animals taking into account that they may have unique metabolic pathways, e.g., for fucoxanthin.

Fucoxanthin is deacetylated into fucoxanthinol by human intestinal epithelial cells and in the digestive tract of mice by lipase and esterase before absorption into the blood circulation [69]. Asai and coworkers showed that fucoxanthinol could be detected in human plasma after 1 week with brown seaweed intake. They conducted a dietary intervention in five healthy subjects with stir-fried wakame (~1 mg fucoxanthin/day), a product consisting of the brown seaweed, *Undaria pinnatifida*. Fucoxanthinol could not be detected at baseline after a 1-week wash-out period without seaweed intake [23]. A mouse study also indicated that fucoxanthinol did not accumulate in any tissue or plasma since fasting levels observed after 1 week of feeding was similar to the background level measured in control mice; despite this finding, the authors concluded that accumulation was taking place in heart muscle but no statistical evidence was presented to substantiate this claim [70]. Half-lives of 3–7 h were observed for fucoxanthinol in most organs with bimodal kinetics and no residual measurable concentration at 24 h after dosing, except possibly for the heart [70]. It has been shown that fucoxanthinol is detectable in plasma 4 h after the administration of a single dose of seaweed extract, but that fucoxanthin is not [22]. The possible reasons are that some components in the seaweed matrix, such as dietary fiber, may inhibit the intestinal absorption of fucoxanthin [66] or that the analytical method was not sufficiently sensitive. Fucoxanthinol could be detected after 24 administrations of kombu, a dried seaweed product consisting mainly of *Laminariacea*, a group of brown seaweeds. Further short-term kinetic studies after fucoxanthin intake in humans are needed in order to evaluate the dose-response kinetics and potential accumulation of fucoxanthinol after a single intake. Interestingly, both human studies mentioned detection of the cis-isomer of fucoxanthinol in plasma after intake of seaweed, but the specific structure was actually not verified.

The bioavailability of fucoxanthinol in humans seems to be lower than that of other types of carotenoids such as lutein, β-carotene, and astaxanthin. In Hashimoto’s study [22], intakes of fucoxanthin around 47 μM resulted in an AUC of fucoxanthinol of below 0.6 μM × h; in comparison, 30% lower intakes of β-carotene, lutein, or astaxanthin resulted in AUCs of 13.6 μM × h, 42.8 μM × h, and 2.26 μM × h, respectively, i.e., considerably higher than for fucoxanthinol [26, 54]. Bioavailability of fucoxanthinol in mouse and rat studies is also lower but not significantly so, compared to the other carotenoids mentioned above [70, 71]. Hashimoto et al. found that fucoxanthinol has a much higher bioavailability in human subjects compared with mice [70], similar to what is observed for other carotenoids [26, 54, 71]. After a single oral dose of 10 ml seaweed extract in 15 human volunteers (only 15% of the dose previously administrated to mice) C_max_ and AUC_(∞)_ were approximately 33% and 46% of those observed in mice, respectively. However, the average elimination half-life of fucoxanthinol excretion was 7 h in humans (4.5 h in mice) and its concentration in urine at 24 h was 7.6 nM (8.2 nM in mice) [22, 70]. The low apparent absorption of fucoxanthin compared to fucoxanthinol in humans may therefore be caused mainly by a higher biotransformation rate into fucoxanthinol.

Some researchers have shown that the food matrix plays a less important role for fucoxanthin than for other carotenoids, but still depending somewhat on fat contents. The lower lipophilicity may limit the intestinal emulsification of fucoxanthinol compared with other carotenoids [23, 72, 73]. Fucoxanthin from seaweed seems to have poor bioaccessibility with a relatively low incorporation of the compound into micelles, explaining its low concentration in human plasma. Deacetylation of fucoxanthin may be caused by enzymes secreted in the human intestines or present within the brush border of the epithelial cells so that biotransformation in the upper gastrointestinal tract is the major cause of the fast appearing peak of fucoxanthinol as well as the very low extent of fucoxanthin absorption. The human enzyme(s) responsible for this degradation still need to be identified.

As already noted, Asai and coworkers mentioned the cis-isomer of fucoxanthinol, but did not provide a detailed identification. Sugawara and coworkers also reported that the metabolite of fucoxanthin observed by HPLC-MS is the *cis*-isomer of fucoxanthinol after incubating with differentiated caco-2 human intestinal cells and plasma from mice [69]. There are also several studies reporting that the *cis*-isomer of fucoxanthinol and halocynthiaxanthin are the metabolites of fucoxanthin in marine animals [74, 75]. However, the latter metabolite has not yet been found in human samples. In studies with several brown seaweeds, the all-trans form was found to be by far more abundant than the cis-forms (9-cis and 13-cis), so it seems most likely that the all-trans form is the usual configuration found in brown seaweeds [76].

### Green seaweed intake biomarkers

#### Astaxanthin

Green seaweeds, such as *Haematococcus*, are the richest natural source of the xanthophyll, astaxanthin (3,3′-dihydroxy-β, β′-carotene-4,4′-dione) [77]. Astaxanthin is found in seaweed as esters where its hydroxyl groups are acylated with fatty acids, while only free forms can be detected in human serum after ingestion; this would indicate that hydrolysis takes place before absorption [25]. Free astaxanthin was found in the gastrointestinal tract (GIT), skin, and blood of humans [24, 25, 78]. Carotenoid bioaccessibility is quite variable among humans and more studies are needed on genetic and acquired variability in carotenoid absorption [79], including the efficiency and inter-individual variability of astaxanthin ester hydrolysis in humans GIT.

Although astaxanthin has been found in blood after intake of green seaweed in human studies [24–26], it is found also in many marine animals [53, 77, 80], due to accumulation in the food chain as a natural lipophilic compound. There are high amounts in the carapace of crustaceans and in the flesh of salmon [24]. Astaxanthin is also present in certain food colorants, e.g., E161. Sources other than seafood are therefore also possible in the diet of humans. In a review of astaxanthin contents in aquatic organisms, levels of 6–25 mg/kg wet weight were found in trout while the levels in microorganisms were 0.001–3.8% of dry weight (10–3800 mg/kg) [80], highest in microalgae. In macroalgae, a high level (0.02% of dry weight, 200 mg/kg) has been reported in *Ulva intestinalis*, a species commonly eaten under the name of green anori while comparable levels in dried shrimp were 0.12% (1200 mg/kg). Within the seafoods, crustaceans and fish would therefore contribute much more than seaweeds to the levels in humans in most diets. While further studies would be needed to compare the bioavailability of astaxanthin from these different sources, the compound will not be considered further here as a biomarker of seaweed intake since astaxanthin is not likely to be robust in a population study.

### Red seaweed intake biomarkers

Red seaweed is another group of seaweeds including several edible species, including dulse (*Palmaria palmata*) from the North Atlantic Ocean and Japanese *nori* (*Pyropia* sp.) from the Pacific. They contain a variety of pigments, including phycoerythrins and carotenoids. Phycoerythrins harvest light energy as part of a protein complex channeling the energy to chlorophyll. No human studies have been performed with red algae to investigate the metabolism of phycoerythrins or their potential use as biomarkers of red seaweed intake.

#### β-carotene

*Nori* contains β-carotene and the content in a sheet of *nori* is the same as that in 10 oranges or in 50 g of carrot. In our search, only one paper described a human study that focused on red seaweed intake. The concentration of serum β-carotene was much higher than normal levels after around 5 months of *nori* intake [28].

However, many fruits and vegetables are rich in β-carotene. Mango, carrot, and green vegetables are all rich in β-carotene [81–84]. Therefore, β-carotene cannot be considered as a candidate biomarker of red seaweed intake in populations ingesting other rich sources of the compound and β-carotene is therefore not considered further here.

### Validation of candidate BFIs

There are totally 7 compounds which can be considered for validation. All of these candidate BFIs for brown seaweeds should be validated according to the procedure previously outlined [17].

#### Phlorotannins

The metabolites of phlorotannins, hydroxytrifuhalol A, 7-hydroxyeckol, C-O-C dimer of phloroglucinol, and phlorotannin-derived candidate biomarkers were detected in hydrolyzed urine in one single-dose intervention without a control group [20]; in addition, some unknown metabolites from an in vitro study were mentioned in this paper. The blood samples were collected at nine time points with the plasma concentration of phlorotannins varying from 0.011 to 7.757 μg/ml; urine samples were collected at three time points with a concentration of phlorotannin ranging from 0.15 to 33.52 μg/ml. Collected urine and plasma samples were stored at − 80 °C until analysis after one and a half years, indicating that the compounds may be stable under these conditions so that measurements are likely to be reliable and feasible; however, multiple measurements of stored samples are needed for a firm conclusion on stability and measurement reliability for the phlorotannin candidate BFIs.

The metabolites of phlorotannins, hydroxytrifurahol A-glucuronide, dioxinodehydroeckol glucuronide, C-O-C dimer of phloroglucinol sulfate, diphlorethol sulfate, and fucophloroethol glucuronide were detected in urine in one crossover study [21] where 24-h urine samples were collected at each time point (weeks 0, 8, 16, and 24) and the concentration of phlorotannin ranged from 0.13 to 522.09 μg/ml. In addition, some unknown or less-significant metabolites in urine were also mentioned in this paper. The collected urine and plasma samples were stored at − 80 °C until the end of the intervention, i.e., for at least 6 months, revealing that the compounds may be stable, but this needs more thorough investigation.

Thus, the phlorotannin candidate biomarkers have short half-lives and may therefore be good short-term markers based on the two human studies. Hydroxytrifuhalol A and C-O-C dimer of phloroglucinol were detected by various types of studies in normal-weight as well as overweight volunteers, indicating that these two compounds are not affected by overweight. Additional studies are still required to verify the stability of these compounds.

The information available for comparing various analytical methods is quite limited so it is difficult to assess the analytical quality of marker analyses in human samples. Additional observational studies are needed to evaluate robustness and dose response. Ideally, a meal study with different levels would be needed for dose-response assessment. Additional studies with other intake assessment methods would also be needed to evaluate reliability of the candidate BFIs. In addition, human studies with several different species of (brown) seaweeds, with cooked seaweed, and with other seaweed preparations are needed to evaluate the influence of food processing on the phlorotannin-derived BFIs. Both existing studies have reported on the presence of the conjugated or enzymatically de-conjugated phlorotannin metabolites in urine. Although phloroglucinol was used as standard in the two studies, quantitative analysis of these compounds would require availability of isotope-labelled standards. Thus, there are no studies available to compare the performance of the analytical methods or to conclude on analytical variability, including accuracy, precision, sensitivity, and specificity of the measurements. Additional studies therefore need to be performed to fully validate the polyphenolic biomarkers of brown seaweed intake.

#### Fucoxanthinol

Fucoxanthinol has been detected in plasma after brown seaweed extract intake in human studies, indicating that it is possible to be considered as a candidate biomarker for estimating intake of seaweeds. Although fucoxanthinol can be detected in some marine mollusks and the concentration there is higher than that in human plasma, the parent compound (fucoxanthin) in brown seaweeds is much higher than that in marine mollusks. Therefore, fucoxanthinol can still be considered a candidate biomarker of brown seaweed intake. Two human studies have reported on fucoxanthinol after brown seaweed intake; one is an oral single-dose study, the other is a 1-week dietary intervention study. The plasma samples were collected at seven time points with the maximum concentration, 44.2 nM, at 4 h after intake of 10 ml kombu extract containing 31 mg fucoxanthin. Thus, fucoxanthinol has a known time response following a single meal study in humans. Additional observational studies and a meal study with different levels are needed to evaluate robustness and dose-response assessment. Collected plasma samples were stored at − 80 °C until analysis in human studies, but no information was provided on the storage time before analysis. Further study of the reliability of fucoxanthinol as a biomarker is needed to validate it. Stability, analytical performance, and reproducibility are also still required to fully validate fucoxanthinol as a biomarker of brown seaweed intake.

## Conclusion

Only a few potentially specific compounds have been found in urine or plasma after intake of seaweeds in human studies, so the number of potential BFIs is small for the groups of brown seaweeds and for the whole group. The few compounds selected as candidate BFIs are hydroxytrifuhalol A, 7-hydroxyeckol, C-O-C dimer of phloroglucinol, diphloroethol, fucophloroethol, dioxinodehydroeckol, and/or their glucuronides or sulfate esters, as well as fucoxanthinol. However, more information is required for their validation, including observational studies to verify robustness, and further meal studies and studies on stability and other aspects of analytical performance are also needed to confirm whether these compounds are specific and feasible for assessing only brown seaweed consumption or possibly consumption of the whole seaweed group. No compounds were found as candidate BFIs for red or green seaweeds. More work is therefore needed to find candidate biomarkers and for their validation before it is possible to objectively evaluate the amount of seaweed consumed by humans.

## Additional file

Additional file 1: **Table S1.** List of literatures reporting specific and non-specific biomarkers for seaweed consumption. Text S1. Validation criteria for biomarkers of food intake. **Figure S1.** Structures of the candidate biomarkers for brown seaweed intake. (DOCX 104 kb)

## Data Availability

Not applicable.
