# Supplementary material for: Biomarkers of seaweed intake
Source: Genes Nutr. 2019 Aug 14;14:24. doi: 10.1186/s12263-019-0648-4 (PMC6694598; doi:10.1186/s12263-019-0648-4)
Supplement: Supplementary file 1 — Table S1. List of literatures reporting specific and non-specific biomarkers for seaweed consumption. Text S1. Validation criteria for biomarkers of food intake. Figure S1. Structures of the candidate biomarkers for brown seaweed intake. (DOCX 104 kb) [file 12263_2019_648_MOESM1_ESM.docx]

**Table S1 List of literature sources reporting specific and non-specific biomarkers for seaweed consumption**

| Food item | Compound investigated | sample type | Discriminating metabolites/Potential candidate biomarkers | Source | Primary Reference |
| --- | --- | --- | --- | --- | --- |
| Brown seaweed | Phlorotannin | Hydrolyzed urine | Hydroxytrifuhalol A, 7-hydroxyeckol, C-O-C dimer of phloroglucinol | Web-database | [1] |
| Brown seaweed | Phlorotannin | Urine | Hydroxytrifurahol A-glucuronide, dioxinodehydroeckol glucuronide, C-O-C dimer of phloroglucinol-sulfate, fucophloroethol glucuronide, diphlorethol sulfate | Web-database | [2] |
| Brown seaweed | Fucoxanthin | Plasma | Fucoxanthinol | Web-database | [3] |
| Brown seaweed | Fucoxanthin | Plasma | Fucoxanthinol | reference list | [4] |
| Brown seaweed | Phlorotannin | Urine | Pyrogallol sulfate | Web-database | [2] |
| Brown seaweed | Phlorotannin | Urine | Phloroglucinol sulfate | Web-database | [2] |
| Green seaweed | Astaxanthin | Plasma | Astaxanthin | Web-database | [5] |
| Green seaweed | Astaxanthin | Serum | Astaxanthin | Web-database | [6] |
| Green seaweed | Astaxanthin | Plasma | Astaxanthin | Web-database | [7, 8] |
| Red seaweed | β-carotene | Serum | β-carotene | Web-database | [9] |

**Text S1 Validation criteria for biomarkers of food intake**

Q1: Is the marker compound plausible as a specific BFI for the food or food group (chemical/biological plausibility)?

Q2: Is there a dose-response relationship at relevant intake levels of the targeted food (quantitative aspect)?

Q3: Is the biomarker kinetics described adequately to make a wise choice of sample type, frequency and time window (time-response)

Q4: Has the marker been shown to be robust after intake of complex meals reflecting dietary habits of the targeted population (robustness)?

Q5: Has the marker been shown to compare well with other markers or questionnaire data for the same food/food group (reliability)

Q6: Is the marker chemically and biologically stable during biospecimen collection and storage, making measurements reliable and feasible (stability)?

Q7: Are analytical variability (CV%), accuracy, sensitivity and specificity known as adequate for at least one reported analytical method (analytical performance)

Q8: Has the analysis been successfully reproduced in another laboratory (reproducibility)?

Fig. S1. Free structures of candidate biomarkers (without sulfate or glucuronide)

**Reference**

1. Corona, G., Y. Ji, P. Anegboonlap, S. Hotchkiss, C. Gill, P. Yaqoob, et al. *Gastrointestinal modifications and bioavailability of brown seaweed phlorotannins and effects on inflammatory markers.* British Journal of Nutrition, 2016. **115**(7): p. 1240-1253.

2. Baldrick, F.R., K. McFadden, M. Ibars, C. Sung, T. Moffatt, K. Megarry, et al. *Impact of a (poly)phenol-rich extract from the brown algae Ascophyllum nodosum on DNA damage and antioxidant activity in an overweight or obese population: a randomized controlled trial.* American Journal of Clinical Nutrition, 2018. **108**(4): p. 688-700.

3. Hashimoto, T., Y. Ozaki, M. Mizuno, M. Yoshida, Y. Nishitani, T. Azuma, et al. *Pharmacokinetics of fucoxanthinol in human plasma after the oral administration of kombu extract.* British Journal of Nutrition, 2012. **107**(11): p. 1566-1569.

4. Asai, A., L. Yonekura and A. Nagao. *Low bioavailability of dietary epoxyxanthophylls in humans.* British Journal of Nutrition, 2008. **100**(2): p. 273-277.

5. Park, J.S., J.H. Chyun, Y.K. Kim, L.L. Line and B.P. Chew. *Astaxanthin decreased oxidative stress and inflammation and enhanced immune response in humans.* Nutrition & Metabolism, 2010. **7**.

6. Okada, Y., M. Ishikura and T. Maoka. *Bioavailability of Astaxanthin in Haematococcus Algal Extract: The Effects of Timing of Diet and Smoking Habits.* Bioscience Biotechnology and Biochemistry, 2009. **73**(9): p. 1928-1932.

7. Mercke Odeberg, J., A. Lignell, A. Pettersson and P. Hoglund. *Oral bioavailability of the antioxidant astaxanthin in humans is enhanced by incorporation of lipid based formulations.* Eur J Pharm Sci, 2003. **19**(4): p. 299-304.

8. Coral-Hinostroza, G.N. and B. Bjerkeng. *Astaxanthin from the red crab langostilla (Pleuroncodes planipes): optical R/S isomers and fatty acid moieties of astaxanthin esters.* Comparative Biochemistry and Physiology B-Biochemistry & Molecular Biology, 2002. **133**(3): p. 437-444.

9. Nishimura, Y., N. Ishii, Y. Sugita and H. Nakajima. *A case of carotenodermia caused by a diet of the dried seaweed called Nori.* J Dermatol, 1998. **25**(10): p. 685-7.
